# Self-directed learning versus traditional didactic learning in undergraduate medical education: a systemic review and meta-analysis

**DOI:** 10.1186/s12909-024-06449-0

**Published:** 2025-01-16

**Authors:** Jasmine Aulakh, Hana Wahab, Christine Richards, Satesh Bidaisee, Prakash V. A. K Ramdass

**Affiliations:** 1 Department of Public Health and Preventive Medicine, St. George’s University School of Medicine, St. George, Grenada; 2 Aureus University School of Medicine, Oranjestad, Aruba

**Keywords:** Self-directed learning, Undergraduate medical education, Medical curriculum, Systematic review

## Abstract

**Background:**

Self-Directed Learning (SDL) is a theory of andragogy in which adult learners take their own initiative to identify and tailor their individual learning process and outcomes. In undergraduate medical education, SDL aims to develop medical students into lifelong learners. This study aims to estimate the overall effectiveness of self-directed learning compared to traditional didactic learning (TDL).

**Methods:**

We performed a systematic review and meta-analysis according to the PRISMA statement. A systematic search was used across PubMed, Scopus, Embase and Google Scholar to identify peer-reviewed articles spanning from January 1, 2014, to May 30, 2024. Key words used were “self-directed learning” AND “undergraduate medical education.” Forest plots were generated with the Open Meta-analyst Software, comparing SDL and TDL.

**Results:**

A total of 2,955 titles and abstracts were screened for eligibility, of which 95 articles met the eligibility criteria for full-text review. Following a more detailed screening, 19 articles met the criteria for inclusion in the systematic review and 14 articles met the criteria for inclusion in the meta-analysis. The systematic review included 2,098 students while the meta-analysis included 1,792 students. The overall mean difference for all studies was 2.399, 95% CI [0.121–4.678], and I^2^ = 98.56%.

**Conclusion:**

Self-directed learning compared to traditional didactic learning is an effective learning strategy in medical undergraduate education and has the potential to aid in students’ learning and improve their cognitive performance. Moreover, SDL nurtures qualities such as autonomy, curiosity, and self-regulation, which are essential for success in the ever-evolving field of medicine.

## Background

Self-Directed Learning (SDL) is a theory that was developed in 1997 by D.R. Garrison who builds on Malcom Knowles theory of andragogy [1]. The premise of SDL emphasizes adult learners to take their own initiative to identify what is needed to learn and is tailored to their own learning process. For undergraduate medical education, SDL is implemented through students taking their own initiative to formulate their individual learning goals. It requires the capacity to identify learning needs, select resources for learning, implement learning strategies and assess the outcomes achieved [2]. Thus, SDL plays a pivotal role in undergraduate medical education, fostering critical thinking, lifelong learning habits, and adaptability, essential qualities for modern healthcare professionals.

In the dynamic landscape of medicine, where knowledge rapidly evolves, traditional didactic approaches alone may fall short in adequately preparing students. Therefore, SDL empowers learners to take ownership of their education, tailoring learning experiences to their needs and interests. This autonomy cultivates curiosity, resilience, and problem-solving skills, all fundamental for navigating the complexities of patient care. Moreover, SDL encourages students to engage with evidence-based practice, fostering a deeper understanding of medical concepts and promoting continuous professional development beyond formal education. By fostering a culture of self-directed learning, undergraduate medical education not only equips students with the foundational knowledge required but also instills the mindset and skills necessary for lifelong learning and excellence in medical practice.

Several schools have incorporated SDL into their curriculum throughout the different phases of medical education, and SDL is increasing in proportion in the curriculum [3]. However, the outcomes differ between SDL and traditional didactic learning (TDL). For instance, a study by Nathaniel et al. [4] showed that SDL was superior to TDL in the formative assessment for a neuroscience module, while Gawad et al. [5] demonstrated that students in the SDL group outperformed students in the TDL group for laparoscopic training [4, 5]. However, a randomized controlled trial between SDL and TDL showed no difference in the accuracy of diagnosing Barret’s esophagus [6].

Thus, the overall effectiveness of SDL in undergraduate medical education is unclear, as studies show diametric outcomes [7, 8]. Therefore, this study aims to evaluate and estimate the overall effectiveness of SDL compared to TDL regarding cognitive performance.

## Materials and methods

### Data sources and search strategy

This systematic review and meta-analysis followed the guidelines and protocols stated in the Preferred Reporting Items for Systematic Reviews and Meta-Analyses (PRISMA) statement [9]. The study protocol was registered in the PROSPERO database, ID CRD42023463524. A systematic search was used across electronic databases including PubMed, Scopus, Embase and Google Scholar to identify peer-reviewed articles. Our search included articles published from 2014 to May 30, 2024. Our search strategy in PubMed, Embase, and Scopus was as follows: (‘self-directed learning’ OR ‘student-directed learning’ OR ‘self-education’ OR ‘independent learning’ OR ‘self-guided learning’) AND (‘medical school education’ OR ‘preclinical medical education’ OR ‘undergraduate medical training’ OR ‘undergraduate medical education’ OR ‘basic medical education’). We limited our search to original articles, and humans. Our search terms used in Google Scholar was “self-directed learning medical education”, with all of the words ‘in the title of the article.’

### Study selection

The Zotero reference management software was used to import citation files from the searched databases, and any duplicate articles were removed. Two investigators (J.A. and H.W.) assessed the relevance of the titles and abstracts of the articles using the eligibility criteria. Any potential full-text articles were further evaluated for inclusion in the review. In cases where discrepancies arose between the authors’ assessment, resolution was through discussion and consensus. The inclusion criteria encompassed experimental (interventional) studies that investigated self-directed learning (SDL) in undergraduate medical education. Specifically, studies were considered eligible if they examined the effectiveness of SDL in comparison to other forms of didactic learning. Studies were included only if they clearly stated that students utilized SDL, even though SDL took various forms (such as problem-based learning) and were referred to by different nomenclature (for instance self-directed teaching and case-based learning). Additionally, SDL needed to be a vital component within a population of undergraduate students pursuing medical education. We excluded meta-analyses, reviews, conference summaries, abstracts, case reports, opinions, letters, and animal studies. Moreover, studies that investigated students’ readiness, preference, satisfaction, and perception of SDL were excluded. Articles were limited to those published in the English language.

### Data extraction, study outcomes and quality assessment

Data were extracted into a standardized data-collection sheet using the following headings: author, date of publication, study site, study design, type of self-directed learning technique, sample size, and findings. Two investigators (J.A. and H.W.) assessed the quality of all included studies using a modified version of the Critical Appraisal Skills Program (CASP) [10],and the overall scores ( with a possible maximum of 14) were recorded. CASP scale is widely used for assessing the quality of each study included in the systematic review and meta-analysis and is based on ranking studies according to the selection criteria, group comparability, and ascertainment of exposure.

### Data synthesis and analysis

Forest plots were generated with the Open Meta-analyst Software, comparing self-directed learning (SDL) and traditional didactic learning (TDL). The mean exam scores for different outcomes were compared between SDL and TDL, and the mean difference in exam scores was plotted. In some instances, in which there were multiple test scores, the average test score was calculated. In cases where there were pretest and posttest scores, we compared the posttest scores between the two groups. In addition, subgroup analyses were done for theoretical and practical SDL.

## Results

### Characteristics of identified studies

**Fig. 1:**
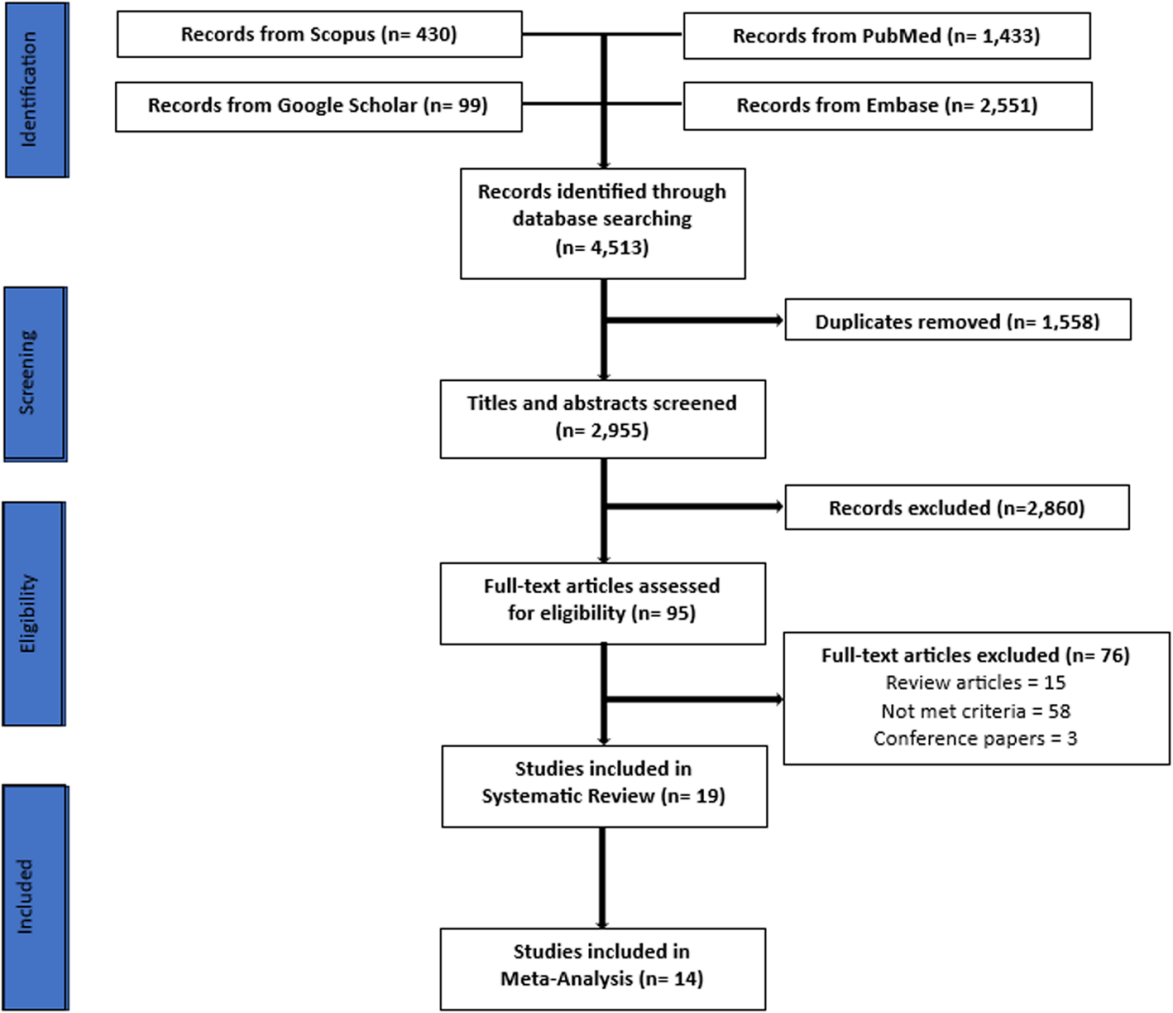
PRISMA flow chart of included studies

As illustrated in Fig. 1, a total of 2,955 titles and abstracts were screened for eligibility, of which 95 articles met the eligibility criteria for full-text review. Following a more detailed screening, 19 articles met the criteria for inclusion in the systematic review and 14 articles met the criteria for inclusion in the meta-analysis. The systematic review included 2,098 students while the meta-analysis included 1,792 students. Students were enrolled in years 1 to 4 of the undergraduate medical curriculum, with most studies analyzed in the meta-analysis having students from year 1. Table 1 shows the characteristics of the studies included in the systematic review and meta-analysis. All study designs were interventional, and two of these were specifically randomized controlled trial. The majority of studies was conducted in India, followed by USA. The CASP scores ranged from 8 to 14, with most studies having a high quality.

**Table 1:** Characteristics of included studies

Study	Study Site	Study Design	Curriculum Year	Sample Size	Number of Students using SDL	CASP Score
Gawad, 2014 [5]	Toronto, Canada	Interventional	First and second	24	12	14
Daly, 2016 [6]	Illinois, USA	Interventional	Second	33	16	11
Peine, 2016 [11]	Germany	Interventional	Third	223	115	12
Raupach, 2016 [12]	Gottingen, Germany	Interventional	Fourth	493	197	12
Allen, 2018 [13]	Kansas, USA	Interventional	Not stated	8	8	8
Diwan, 2017 [14]	Gujarat, India	Interventional	First	26	13	9
Lian, 2017 [7]	Perth, Australia	Interventional	Not stated	30	10	10
Nathaniel, 2018 [4]	South Carolina, USA	Interventional	First and second	105	53	9
Anantharaman, 2019 [15]	Southern, India	Interventional	Second	53	53	8
Andersen, 2020 [16]	Copenhagen, Denmark	Interventional	Not stated	15	15	10
Chakraborty, 2022 [17]	Burdwan, India	Interventional	First	200	100	12
Chimmalgi, 2022 [18]	Kerala, India	Interventional	First	150	150	9
Palve, 2022 [19]	Puducherry, India	Interventional	Not stated	250	125	11
Akbari, 2023 [20]	Ontario, Canada	Interventional	Third and fourth	27	18	12
Biswas, 2024 [21]	Kolkata, India	Interventional	First	60	30	10
Gayathri, 2024 [22]	Hyderabad, India	Interventional	First	150	75	13
Kashou, 2024 [23]	Worldwide	Interventional	Not stated	91	46	10
Roy, 2024 [24]	West Bengal, India	Interventional	First	200	100	8
Surapaneni, 2024 [25]	Chennai, India	Interventional	First	74	148	12

### Types of SDL

As shown in Table 2, two main categories of Self-Directed learning (SDL) were identified: [1] theoretical, whereby students gained knowledge on various subjects and were then tested on formative and summative assessments; and [2] practical, whereby students engaged in hand-on experience by performing various procedures or exhibiting certain skills. SDL through theoretical approach consisted of clinical encounter cards, audio visual presentation, computer assisted SDL, team-based SDL, combined didactic-SDL teaching, online-SDL, and case-based SDL. SDL through practical approach consisted of clinical skills, in particular ultrasound guided insertion of PIVC, laparoscopic skills, and anatomical practical performance.

**Table 2:** Description of SDL types

Study	SDL Type	Findings
Gawad, 2014 [5]	(Practical) Laparoscopic Skills	Supervised learning and practice compared to self-directed approach in Stimulation based training curriculum (STC) with laparoscopic skills. Supervised training group outperformed SDL group in peg transfer task.
Daly, 2016 [6]	(Theoretical) Automated Presentation with Audio	In-classroom training compared to SDL training in predicting Barret’s Esophagus through NBI Imaging. No significant difference identified.
Peine, 2016 [11]	(Theoretical) Self-instructed learning	Self-directed learning format compared to Instructed learning format. The SDL group reached higher scores compared to non-self-instructed group.
Raupach, 2016 [12]	(Theoretical) ECG Interpretation	Three types of approaches compared: SDL, lectures, and small-group peer teaching on students’ skills retention. The study concluded that compared to SDL, more intensive teaching did not increase performance levels.
Allen, 2018 [13]	(Theoretical) Colorectal polyps	narrow band imaging in diagnosing colorectal polyps using the NICE Criteria
Diwan, 2017 [14]	(Theoretical) CBL	Study designed to introduce an SDL approach using Case-Based Learning (CBL) and to evaluate its impact. Study concluded that CBL can be used along with lectures to strengthen traditional methods of learning.
Lian, 2017 [7]	(Practical) Ultrasound Guided insertion of PIVC	Three types of approaches compared: no education, self-guided internet-based education and normal face-to-face traditional education in Ultrasound guided insertion of peripheral IV vascular cannulae (PIVC). The traditional face-to-face teaching group performed significantly better than the other groups.
Nathaniel, 2018 [4]	(Theoretical) Clinical case Learning	The study assessed the outcome on SDL teaching compared to no SDL teaching in Neuroscience. Significant improvement in test scores identified in the SDL group.
Anantharaman, 2019 [15]	(Theoretical) PBL	Study designed to assess the effect of a two-month problem-based learning course on Self-Directed learning. The study concluded that a short-term problem-based learning course positively influences self-directed learning.
Andersen, 2020 [16]	(Practical) Simulation based mastoidectomy	Study designed to understand effects of SDL in simulation-based training of mastoidectomy. The study concluded that SDL promoted cognitive engagement and motivation in learning tasks to facilitate self-regulated learning.
Chakraborty, 2022 [17]	(Theoretical) Physiology	Traditional lecture-based classes were compared to SDL sessions. Students who received SDL sessions performed much better in post-test sessions compared to students who received lecture-based classes.
Chimmalgi, 2022 [18]	(Theoretical) Online modules	Study designed to determine the effectiveness of SDL modules. The study concluded that using SDL in a blended approach showed significant improvement in students’ academic performance.
Palve, 2022 [19]	(Theoretical) Cardio-respiratory physiology	Students divided into SDL group and normal didactic lectures were given assessments post lecture. The SDL group showed significantly higher scores in comparison to the didactic learning session group.
Akbari, 2023 [20]	(Practical) Plastic Surgery	E-learning module was created, and two cohorts were recruited. The interventional cohort completed their plastic surgery rotation with the use of the module, while the control cohort did not. The study concluded that the Interventional group scored significantly higher than the control group and displayed more confidence during their plastic surgery clinical rotation.
Biswas, 2024 [21]	(Theoretical) Anatomy of the liver	To evaluate learning outcomes after exposure to SDL, as well as the traditional demonstration/ prosection to compare the effectiveness of SDL with the conventional method of teaching by demonstration/ prosection in learning the anatomy of the liver
Gayathri, 2024 [22]	(Theoretical) Muscle anatomy	To assess SDL as a new teaching learning method in gross anatomy
Kashou, 2024 [23]	(Practical) ECG interpretations	The study aimed to assess the effectiveness of web-based, self-directed interventions in improving ECG interpretation skills. The study concluded that self-directed interventions markedly enhanced ECG interpretations skills.
Roy, 2024 [24]	(Practical) Anatomy Practical Performance	Study designed to compare students learning through SDL approach to students learning through traditional methods. The study revealed that the SDL approach to learning anatomy positively impacted students’ academic performance compared to students being taught using conventional methods.
Surapaneni, 2024 [25]	(Theoretical) Bronchial asthma, coronary artery disease, chronic obstructive pulmonary disease	Self-directed, Problem-oriented, Lifelong learning, Integrated Clinical case Exercise (SPLICE) modules to promote critical thinking skills
*SDL *Self-Directed learning, *DT *Didactive Teaching, *NBI *Narrow Band Imaging, *DT/SDL* Didactic Teaching and Self-Directed Learning, *OCLI * Oddi Continuing Learning Inventory, *EBM *Evidence Based Medicine, *STC *Stimulation based Training Curriculum, *PIVC* Peripheral IV Vascular Cannulae (PIVC), *CBL *Case-Based Learning, *PBL *Problem-Based Learning, *LPB *Lecture Based Problems		

### SDL versus TDL

**Fig. 2:**
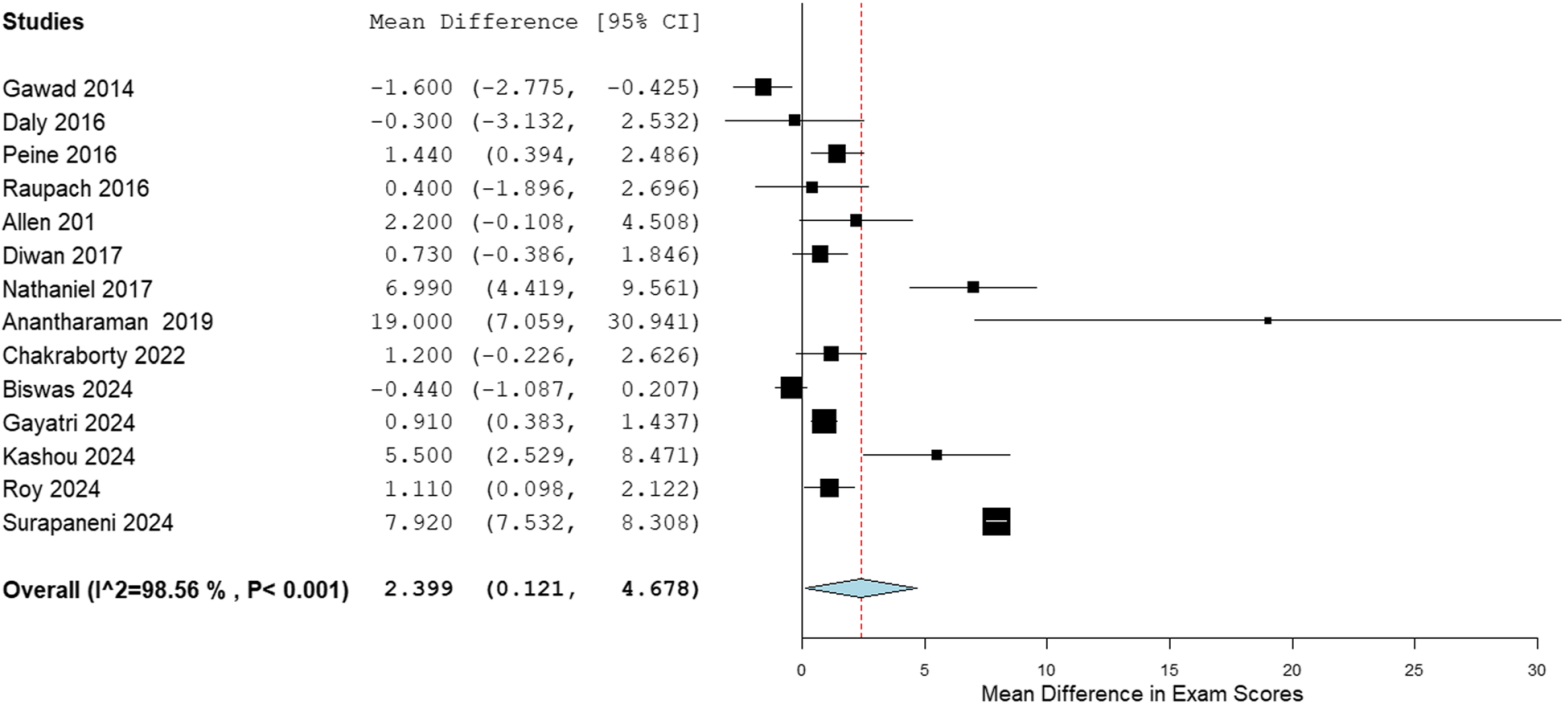
Forest plot all studies

**Fig. 3:**
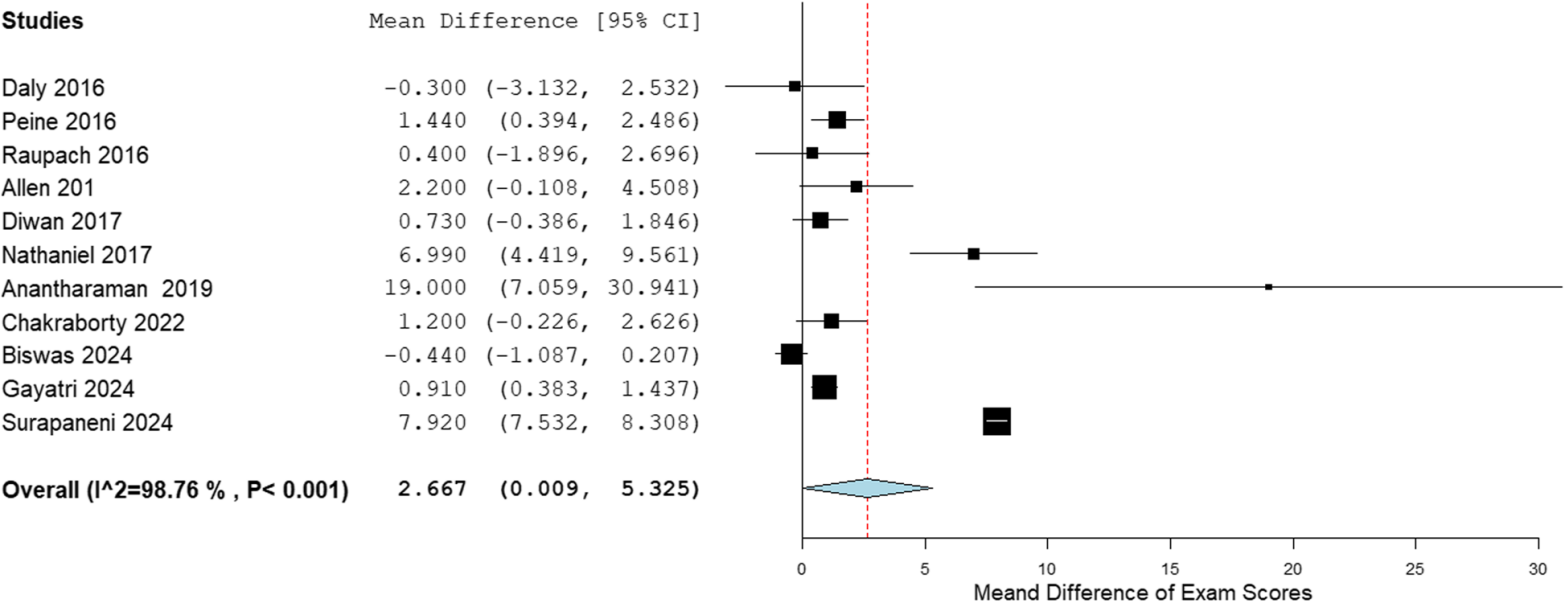
Forest plot theoretical

**Fig. 4:**
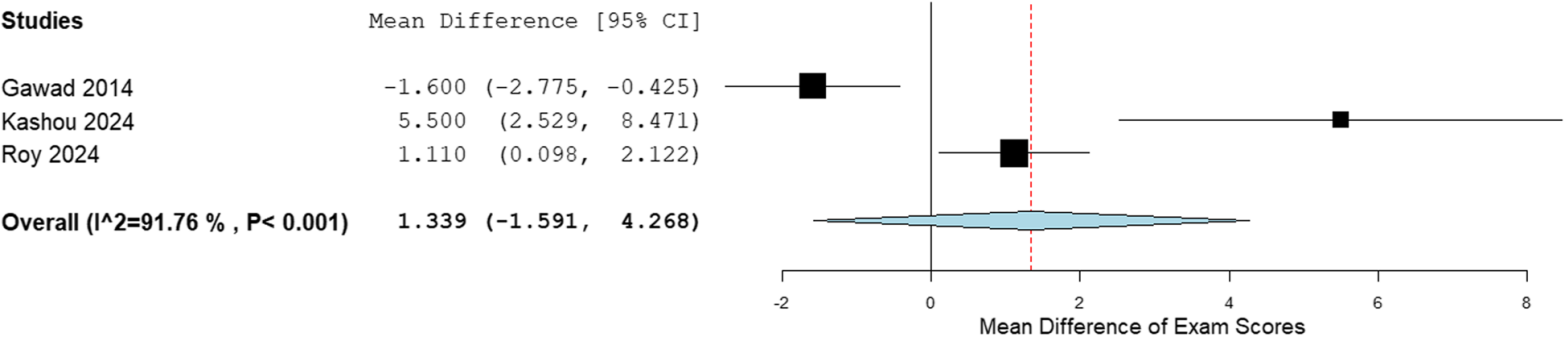
Forest plot practical

Table 3 shows the mean and standard deviation (SD) of the exam scores for SDL and TDL. The pooled analysis for all studies is shown in the forest plot in Fig. 2. Of the 14 studies in the meta-analysis, 11 studies showed that the mean exam scores was higher for SDL when compared to TDL, whereas only 3 studies showed better performance in exam scores for TDL. The overall mean difference for all studies was 2.399, 95% CI [0.121–4.678], *p* < 0.001, and I^2^ = 98.56%. The mean difference in exam score for these studies ranged from − 1.6 [5](favoring TDL) to 19.0 (favoring SDL) [15]. The overall mean difference in exam scores for the subgroup analysis for theoretical SDL was 2.667, 95% CI [0.009–5.325], and I^2^ = 98.76%, shown in Fig. 3. The overall mean difference in exam scores for the subgroup analysis for practical SDL was 1.339, 95% CI [−1.591–4.268], and I^2^ = 91.76%, shown in Fig. 4.

**Table 3:** Mean exam scores of SDL versus TDL

Study	SDL # of Students	SDL Mean	SDL SD	TDL # of Students	TDL Mean	TDL SD
Gawad, 2014 [5]	10	5.1	1.4	12	6.7	1.4
Daly, 2016 [6]	16	57.2	4.1	17	57.5	4.2
Peine, 2016 [11]	54	15.81	2.81	55	14.37	2.76
Raupach, 2016 [12]	136	80.7	8.9	77	80.3	7.8
Allen, 2018 [13]	8	80	2.2	8	77.8	2.5
Diwan, 2017 [14]	13	6.88	1.59	13	6.15	1.3
Nathaniel, 2018 [4]	53	80.67	3.65	52	73.68	8.74
Anantharaman, 2019 [15]	36	214	29	53	195	27
Chakraborty, 2022 [17]	100	69.33	5.65	100	68.13	4.58
Biswas, 2024 [21]	30	8.53	1.17	30	8.97	1.38
Gayathri, 2024 [22]	75	8.41	1.83	75	7.5	1.44
Kashou, 2024 [23]	212	66.7	15.5	209	61.2	15.6
Roy, 2024 [24]	100	11.37	3.7	100	10.26	3.6
Surapaneni, 2024 [25]	74	26.69	0.71	74	18.77	1.55
*SDL *Self-directed learning, *TDL *Traditional didactic learning, *SD *Standard deviation

## Discussion

Our analysis shows that self-directed learning (SDL) outperforms traditional didactic learning (TDL) in exam scores for medical undergraduate education. Even though the attributes of the studies compared were diverse, ranging from diagnosis of Barrett’s esophagus [6] to electrocardiography interpretation [12, 23] to laparoscopic skills, the overall mean score on knowledge application, cognitive performance, and acquired surgery skills etc., achieved by the students was higher for SDL than TDL. This is especially true for theoretical SDL as the students performed better in 9 out of the 11 studies compared to TDL.

While SDL may not be as beneficial as traditional in-classroom didactic lectures for selected cognitive competencies or knowledge acquisition for certain subject areas, there is a proven advantage for its role in areas such as neuroscience [4] and endocrine pharmacology, specifically learning the diabetes mellitus diagnosis guideline and treatment algorithm [11]. Moreover, Andersen et al. demonstrated that SDL is a more effective learning strategy when compared to conventional TDL for improving students’ skills in mastoidectomy [16]. Likewise, the study findings from Roy et al. have shown that there was a significant improvement in anatomical practical performance for students in the SDL compared to students in the TDL group [24].

Moreover, a rise in e-learning and online courses demanded SDL an essential skill needed for medical education and life-long learning [26]. As the number of online education opportunities continue to grow and with the advent of rapidly growing new technologies, such as Artificial Intelligence (AI), SDL has proven to be a necessary component of education, including undergraduate medical education. Successful SDL requires students to be highly motivated, team players, with problem-solving and time management skills. Learners should also be equipped with skills to source and filter relevant information from quality sources [27]. These characteristics are prevalent among medical students and may therefore explain the finding of this study. According to the AAMC (Association of American Medical Colleges) competencies of successful applicants to undergrad medical programs include communication skills, social skills, resilience and adaptability, critical thinking, quantitative reasoning and scientific enquiry; skills which better equip students for successful SDL opportunities [28].

Regarding specific competencies or knowledge acquisition, there is a proven advantage for its role in areas such as surgical techniques or interpretation of test findings. For instance, Akbari et al. demonstrated that SDL is an effective learning strategy when compared to TDL for improving students’ knowledge for certain surgical topics [20]. In addition, Daly et al. showed that SDL and in-classroom lectures had similar accuracy for diagnosing Barret’s esophagus [6]. Furthermore, SDL may help students develop self-reliance on preparing for the challenges that are encountered with the fast-paced evolution of medicine and health care.

This review demonstrates several strengths. First, it used a comprehensive systematic search encompassing articles from the field’s inception up to the review date. Furthermore, it utilized internationally recognized criteria to assess the quality of the studies included. However, the conclusions of the study should be considered within context of the ensuing limitations. The methodologies and sample sizes of the reviewed studies varied widely with five of the sixteen studies being pilot studies with small sample sizes. Moreover, the application of the Self-Directed Learning (SDL) approach was confined to a limited subset of topics within undergraduate medical curricula, potentially skewing perceptions of its effectiveness to those specific areas. Further research is warranted to explore the efficacy of SDL across a broader spectrum of topics, incorporating larger and more diverse samples from various universities. Additionally, the studies reviewed represented a global range of countries with potentially differing pedagogical approaches and cultural backgrounds which could have influenced attitudes towards, and perceptions of SDL. Future research should explore the correlation between student traits and success rates when utilizing SDL and determine the optimal level at which to integrate SDL into undergraduate medical education.

However, our analysis was limited by the heterogeneity of study designs used, varying sample size, methodology and outcome measures which may affect the reliability and generalizability of the findings. In addition, our analysis included studies that were conducted from four different years of the medical curriculum. Thus, students were at various stages of learning and training. However, despite this diversity in education level, SDL and TDL were compared within year and not between different years. Assessing the risk of bias in individual studies and its potential impact on the overall results is crucial in reviewing and reporting on analyses conducted. Additionally, publication bias as a tendency for studies with positive results to be published, may have led to an overestimation of the effectiveness of SDL if unpublished studies with negative results are not included in the analysis. SDL interventions can also vary widely in terms of format, duration, content, and delivery methods. This variability may make it challenging to draw definitive conclusions about the effectiveness of SDL.

Notwithstanding, SDL in undergraduate medical education can have several implications for both medical education practices and future research. Medical educators can use the findings to inform the design and implementation of SDL interventions within undergraduate medical curricula. Understanding which aspects of SDL are most effective can help educators tailor curricular components to enhance student learning outcomes. Educators can explore different pedagogical approaches to support SDL, such as incorporating technology-enhanced learning tools, providing mentorship or guidance, and fostering a supportive learning environment that promotes autonomy and self-regulation. For instance, SDL had a major contributory role during times of recent global crisis with the Corona virus disease 19 pandemic, in which a sudden transition to online courses necessitated an increasing need to develop SDL skills [20]. Thus, when used appropriately and strategically as a learning strategy, SDL can be beneficial to medical education and should be incorporated into the curriculum. Moreover, SDL has integrated different learning formats such as problem-based learning and case-based learning.

Given the importance of assessment in guiding and evaluating SDL, educators may need to develop and implement appropriate assessment strategies that align with the goals and objectives of SDL initiatives. This could include formative assessments, self-assessment tools, and peer evaluation methods. Future research should explore how SDL initiatives can be tailored to meet the diverse needs of medical students, including those from underrepresented backgrounds or with varying learning styles. This could involve culturally responsive pedagogical approaches and addressing barriers to participation in SDL activities.

## Conclusion

Our analysis indicates that self-directed learning (SDL) compared to traditional didactic learning (TDL) is an effective learning strategy in medical undergraduate education and has the potential to aid in students’ learning and improve their cognitive performance. While didactic teaching provides a structured framework for delivering information, SDL empowers students to actively engage with and construct their knowledge, leading to deeper understanding and retention of information. Moreover, SDL nurtures qualities such as autonomy, curiosity, and self-regulation, which are essential for success in the ever-evolving field of medicine.

## Data Availability

Not applicable.

## Abbreviations

SDL: Self-Directed learning
TDL: Traditional Didactic learning
DT: Didactic Teaching
NBI: Narrow Band Imaging
DT/SDL: Didactic Teaching and Self-Directed Learning
OCLI: Oddi Continuing Learning Inventory
EBM: Evidence Based Medicine
STC: Stimulation Based Training Curriculum
PIVC: Peripheral IV Vascular Cannulae (PIVC)
